# Why They Are Different: Based on the Burden of Disease Research of WHO and Institute for Health Metrics and Evaluation

**DOI:** 10.1155/2018/7236194

**Published:** 2018-04-23

**Authors:** Seok-Jun Yoon, Young-Eun Kim, Eun-Jung Kim

**Affiliations:** ^1^Department of Preventive Medicine, Korea University, Seoul, Republic of Korea; ^2^Department of Nursing, Pyeongtaek University, Pyeongtaek, Republic of Korea

## Abstract

**Objectives:**

We traced the methodology of measuring the burden of disease of IHME and WHO in detail and we would like to present various perspectives on the aspects that can be acceptable in Korea or not.

**Study Design:**

We investigate the methodology and the major outcomes of the studies of burden of disease and show the direction of our future research. We studied and compared WHO's and IHME's outcomes in aspect of the data source, methodological differences, and the interpretation ways.

**Results:**

Despite the in-depth review, there was “black box” that could not be explained specifically. But there were some estimations and using of data from developed countries which had well-developed population polls. In addition, using DisMod-MR for metaregression of IHME was different from WHO's DisMod-2.

**Discussion:**

It will be necessary to secure the validity of the claim data in order to trace the accuracy of the disease diagnosis. At last, the accuracy of the data used to construct the disease burden survey system should be promoted. To this end, we propose to introduce a cause-of-death estimation system, linking the cause-of-death report and the health insurance claiming system with the electronic medical records that the hospital produces.

## 1. Introduction

Predicting the burden of disease is a key foundation for building a platform for planning and prioritizing health policies and evaluating the effectiveness of intervention programs. In addition, the information regarding effects of intervention, social acceptability, side effects, cost-effectiveness, and total cost is necessary for the policy-making stage. It is the most optimal information to be measured by using accurate data. However, if it is difficult in reality, predicting the burden of disease through proper data and modeling can be a next step.

Research of the burden of diseases has been actively carried out under the leadership of the Institute for Health Metrics and Evaluation (IHME). Christopher Murray, the founder of the institute, joined the WHO research cowriter in 1995 as part of the WHO World Development Report 1993 and joined as a researcher with his colleague, Alan Lopez, the Global Burden Disease estimation team. Since then, he had been promised research funding from WHO for independent research and come back to Harvard University, but the offer was rejected by WHO and became a founder of the IHME, funded by the Bill and Melinda Gates Foundation in 2007. In an interview with the New York Times, Murray said that WHO's research has lost fairness due to the pressure of the funding member states and will open a new chapter in disease burden research through IHME. In 2017, IHME will receive $ 279 million in funding from the Bill and Melinda Gates Foundation for 10 years, the largest since it was founded.

Looking at the purpose of establishing this institute is to find the best strategy for creating a healthy world. For this, they provide information for decision-making by measuring health levels, tracking health program outcomes, seeking ways to maximize health system impacts, and developing innovative measurement systems. Ultimately, this suggests seeking the way of improving global health. IHME presents a specific and comprehensive methodology for predicting disease burden and presents the burden of disease due to disability and premature death at international, national, and local community level. The methodology uses complex mathematical models and is integrated into a single framework based on specific assumptions [1].

In order to estimate the burden of disease, it is available in a number of countries and has relatively accurate population data. In developed countries, relatively accurate disease burden is measured based on biomass statistics and disease registration data. However, in the case of data constructed in underdeveloped countries, it is a problem in terms of reliability and enrollment rate. Such problems cause errors in mathematical modeling and therefore it is difficult to predict the exact disease burden. In this case, there are somewhat unacceptable assumptions, such as replacing figures from developed countries or applying modeling in developed countries as it is.

## 2. Objectives

In this article, we traced the methodology of measuring the burden of disease of IHME and WHO in detail and we would like to present various perspectives on the aspects that can be acceptable in Korea or not.

## 3. Results

IHME officially announced the International Burden of Disease Survey through “Global Burden of Disease 2010,” a special edition of Lancet in 2012 [2].

The main results show that infectious diseases, maternal and child mortality and diseases, and malnutrition deaths and diseases have decreased worldwide, but the burden of disease has increased in noninfectious diseases (cancer, heart disease, etc.) in youth [2]. Life expectancy has increased by more than 10 years, but YLD has also been reported to increase compared to the 1970s [2].

**Figure 1:**
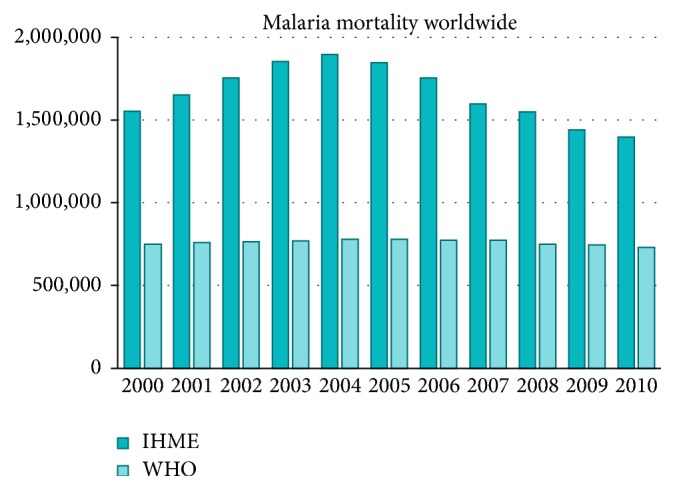
Mortality of malaria (IHME versus WHO).

However, there is room for controversy when examining the characteristics of individual diseases and measures. For example, the number of deaths due to malaria of WHO was 655,000, which is half that of IHME (1,240,000) [3] (Figure 1).

These results led to controversy over the effectiveness of the WHO's ongoing efforts to combat malaria and faced many criticisms. With or without the recognition of criticism, IHME has decreased to 855,000 in the GBD 2013 report without any comments [4, 5].

Also, the mortality rate reported by IHME has been controversial. IHME reported 817,000 deaths between the ages of 5 and 15, and this figure was borrowed from the UN report [6]. In fact, when we look at the UN report data, the deaths are 164 million [7], which is not consistent with the IHME's report.

Therefore, the WHO did not recognize the findings of the IHME. Only data reported by Lozano et al. (2012), which confirmed the source and accuracy of the data, were made available for retrieval from WHO “Global Health Observatory.” The global health observatory is an initiative of the WHO to share data through their website on global health, including statistics by country and information about specific diseases and health measures.

In this way, IHME's methodology for measuring burden of disease has an unclear stage called “black box step.” In particular, only the Bayesian metaregression analysis and DisMod-MR were used to explain the YLD measurement method that should estimate the morbidities and the patients, but no specific method is described [2]. WHO requested sharing of data processing methods, but was informed of the inability to do so. For this, WHO researches were recommended to avoid collaborative work with IHME [8].

After a lot of controversy, IHME updated its data in 2015 by publishing “Global Burden of Disease 2013.” The main improvements are summarized as follows: The cause of disease and the sequelae list were confirmed through a comorbid disease prediction model. We analyzed the severity of chronic obstructive pulmonary disease (COPD) and diabetes mellitus (DM) and measured the burden of disease on the basis of sequelae. Patients were divided according to the severity of liver cancer, hepatitis B or C, or alcoholic epilepsy through meta-analysis. In addition, the code of each disease was subdivided to present the disease in more detail. We improved the DisMod-MR and developed the predictive model through the DisMod-MR 2.0 version. Disability weights were also newly surveyed and announced by each country [4].

As a result, IHME presented the burden of disease according to 301 diseases and traumas, 2,337 sequelae, and 79 risk factors in 188 countries [4]. In detail, in developed countries, an age-standardized mortality of cardiovascular disease and cancer declined, and in developing countries child diarrhea disease, lower airway infections, and neonatal deaths decreased [4]. YLD was also estimated to decrease with age standardization, and the decrease rate of YLL was greater than that of YLD [4].

In the report of “Global Burden of Disease 2013,” it should be noted that DisMod-MR is an improvement [4]. First, The DisMod-2 used in the WHO-published disease burden study estimated the age of onset and the duration of the disease by providing a single-digit predictive value. For the purpose of “assumption,” a somewhat flexible model is needed, but DisMod-2 is given as a single value. There is criticism that there must be a need for a method to adjust various covariates. So, they need some prediction models using meta-analysis, Bayesian model, and regression model [4].

Therefore, IHME developed DisMod-MR, which is completely different from DisMod-2 used in previous GBD research. Bayesian analysis can provide more flexible predictive values and estimate uncertainty to provide an uncertainty interval [4]. In addition, meta-analysis can be used to specifically adjust international and regional values, and regression analysis can also adjust for covariates such as age and comorbidity diseases [4].

GBD 2013 uses DisMod-MR version 2.0, which improves computational speed and improves cascade program accessibility, which is used to calibrate fitness when applying the national model as an international model (Table 1) [4].

In one study, they compared the methodology and the way how IHME and WHO estimated the risk of diarrhea and pneumonia due to childhood mortality [5]. In terms of data sources, the IHME estimates the burden of diseases using data for enrollment rate of 60% or more and includes all the results using verbal autopsy death data. And they included journals on disease burden measurement in systematic literature review [5]. On the other hand, WHO selected data more conservatively. That is, only biomarkers with more than 80% registration rate were included in the systematic review of the literature and they included oral autopsy studies only in countries with high mortality rates and low enrollment rates [5]. In the case of data processing, IHME considered garbage code at all childhood ages, while WHO reassigned the cause of death to infants aged 1 to 59 months [5]. So the mortality of children of IHME was higher than WHO (Table 2).

## 4. Discussion

We would like to consider the future direction of Korea's disease burden research in relation to past and present work of IHME. The first is the aspect of the necessity of “estimation.” The reason for IHME estimation is because of lack of data sources. In other words, it is difficult to measure various sequelae because there are no biometric statistics in which 60% of the subjects are registered. In the case of Republic of Korea, however, it is possible to derive the national epidemiological index through the claim data of the National Health Insurance. In addition, since it is possible to estimate the various sequelae presented by IHME using a subdiagnosed disease or a treatment code, a prediction process through meta-analysis is not essential. The IHME warned of the danger of unmet need for the use of these medical claims data. Currently, the unmet medical status in Korea is 14.89% in 2011, 16.38% in 2012, and 17.64% in 2013 [9], which is much higher than the registration rate of 60% proposed by IHME. Therefore, it is considered to be superior in terms of reliability of data compared with the estimation. In addition, since the cohort data on major diseases (diabetes, cancer, disability, etc.) are constructed by each academic society, it can be judged as a good environment to measure disease burden without estimation process.

Second, we have to decide whether to use WHO methods as usual or not. WHO estimates the age of onset and the duration of diseases using DisMod-2. This estimate is limited because it requires a one-to-one correspondence with the disease. So we cannot analyze this considering comorbidity or severity. In this case, we can consider whether this work should be estimated through DisMod-2 or not. It can be indirectly grasped through the analysis of hospitalization period and age of onset using National Health Insurance claims data. At this time, it will be necessary to secure the validity of the claim data in order to trace the accuracy of the disease diagnosis.

We would like to suggest some policies for the measurement of the burden of disease. First, we propose to establish a disease burden investigation system. It examines the status of data collection in social insurance countries such as the UK and Germany, identifies the international situation, and publishes the status of the disease burden periodically by including these data as national statistics. It is used as basic data of various diseases researches and as evidences data for policy making.

Second, the accuracy of the data used to construct the disease burden survey system should be promoted. Currently, there is uncertainty in utilizing the Korean cause-of-death statistics and insurance claims data. The reason for this is that the leading cause of death is divided unclearly and there are many diagnoses that cannot be used as cause of death. In addition, due to the medical routine or provision of diagnostic codes for prevention insurance cutback, there might be possibilities of inaccurate diagnosis. To this end, we propose to introduce a cause-of-death estimation system, linking the cause-of-death report and the health insurance claiming system with the electronic medical records that the hospital produces. Using this system, we can get not only the cause of death of diagnosed diseases as a simple ICD-10 code but also the individual history of medical care more accurately. Kanta, the e-Health service in Finland, is a good example of this. Through this system, not only the prescription of medication data, but also the information on the use of the primary medical clinic service data could be integrated comprehensively. It is also evaluated as effective and efficient in providing more in-depth and accurate disease-related epidemiologic data by providing this information to the medical staff as well as to the patient concerned.

## Figures and Tables

**Table 1:** Update of DisMod-MR.

DisMod-2	DisMod-MR	DisMod MR 2.0
(i) Parametric assumption with single number (ii) Needing more flexible model for estimation (iii) Adjusted covariate variables (iv) Lack of meta-analysis, Bayesian inference, regression	(i) Bayesian analysis (a) Flexible model (b) Markov Chain Monte Carlo for hierarchical model (c) Availability of uncertainty interval (ii) Meta-analysis: global, regional fit (iii) Regression: adjusted age, comorbidity, etc. (iv) UI of “p X RR” (v) Adjusting the local fit using global fit with cascade	Faster than DisMod-MR of calculation

**Table 2:** Methodology of IHME and WHO.

	IHME	WHO
Modeling process	(1) Data identification (2) Data processing (3) Cause of death ensemble model (CODEm) (4) Proportional model for dividing hierarchical diseases (5) DisMod-MR model for estimation deaths (6) CoDCorrect for scaling cause-specific mortality fraction to sum to 1	(1) Data source identification (2) Data processing (3) Use of one of 3 models to estimate neonatal and postneonatal mortality separately, depending on mortality level, disease profile, and data availability (4) Development of a disease-specific model (5) Adjusting death to sum to the total mortality

Data source	(i) VR (vital registration): over 60% registered data only (ii) VA (verbal autopsy): including all the related paper (iii) Hierarchical disease: systematic review, disease burden estimation paper	(i) VR (vital registration): over 80% registered data only (ii) High mortality & low registered rate: SR with VA (iii) Including regarding intervention effects

Data processing	Considering garbage code of cause of death through all ages	Considering garbage code of cause of death only newborn

## Data Availability

The data used to support the findings of this study are available from the corresponding author upon request.
